# Polyphenol-Rich Foods for Human Health

**DOI:** 10.3390/nu12123738

**Published:** 2020-12-04

**Authors:** Patrizia Restani

**Affiliations:** Department of Pharmacological and Biomolecular Sciences, Università degli Studi di Milano, via Balzaretti 9, 20133 Milano, Italy; patrizia.restani@unimi.it; Tel.: +39-02-5031-8350

In recent decades, foods rich in polyphenols have received great interest from researchers, who have performed numerous studies in in vitro and animal models and clinical trials. In parallel, food industries have spent many resources in the formulation of functional foods and food supplements that could provide the consumer with an enriched source of these molecules. The consumer has certainly appreciated the expansion of the market for products of “natural” origin, as they consider them (often erroneously) safe by definition. Polyphenols, compounds from vegetable origin with no nutritional value, have long been considered “functional ingredients”, i.e., positive for human health. On the other hand, given that polyphenols are not strictly necessary for the physiological functions of the body, as in the case of vitamins and minerals, the scientific committees often raise concerns on the validity of the studies from which any recommendations for intake derive. Among the few claims allowed for polyphenols, there is that published by the EFSA (Commission Regulation (EU) 432/2012) relating to olive oil: “Olive oil polyphenols contribute to the protection of blood lipids from oxidative stress. The claim may be used only for olive oil, containing at least 5 mg of hydroxytyrosol and its derivatives (e.g., oleuropein complex and tyrosol) per 20 g of olive oil. In order to bear the claim information shall be given to the consumer that the beneficial effect is obtained with a daily intake of 20 g of olive oil”.

Among the various properties described, phenolic compounds possess the ability to neutralize free radicals; it is therefore thought that their main role may consist in counteracting oxidative stress at the cellular level, and the health claim allowed for olive oil supports it. The antioxidant effect, which is the activity in counteracting the deleterious effects of free radicals, can modulate many of the risk factors responsible for: (1) chronic degenerative diseases, such as tumor diseases, cardiovascular disorders, metabolic syndrome, and dementia; (2) degenerative physiological processes, such as aging.

In more recent times, several authors have published data in support of other beneficial activities related to polyphenols [1]: anti-inflammatory activity, inhibition of tumor cell proliferation, inhibition of cholesterol absorption, modulation of some enzymatic activities that fall within in the mechanisms of stimulation or inhibition of cellular metabolic processes. On these activities, however, apart from the anti-inflammatory activity [2], there are still many uncertainties. In general, polyphenols are ideally considered and often even advertised as “protectors” easily obtainable with the diet. Numerous authors confirm this hypothesis, but, as mentioned above, most of the studies derive from experimental assays performed in vitro or in animal models, from which extrapolation to humans is difficult. In vitro studies use generally purified molecules at high concentrations unreachable with the dietary intake; even a compound with very high antioxidant activity or other biological activity in vitro could have little or no effect if in vivo it does not reach the target tissue at a sufficient concentration.

Furthermore, the metabolic processes that polyphenols undergo in the human body are not yet fully known, even taking into account that they are consumed with complex matrices (such as fruit and vegetables) or foods in combination. For example, the study by Serafini and co-workers [3] showed how the addition of milk can drastically reduce the antioxidant activity of green and black tea. The effect on the human body may be different from that observed in the tissues in culture, where the molecules are poorly biotransformed. The paper published in 2013 by Stockley and co-workers [4] has collected and critically evaluated the studies relating to the bioavailability of the various classes of polyphenols taken with the diet and in particular with wine. The review led to the conclusion that there are still many critical issues to be studied before being able to reliably associate the presence of one or more classes of polyphenols in foods with the health effects on the cardiovascular system (the most described one) or on other systems/organs.

One of the few certainties lies in the numerous epidemiological studies, where the consumption of fruit and vegetables is considered critical for the positive effects of the diet on health. In confirmation of this, the Mediterranean diet is now universally known thanks to its inclusion by UNESCO in the intangible heritages of humanity. This Special Issue of *Nutrients* collects new information on the role of polyphenols in human health considering the different classes of molecules, their bioavailability, the synergy between the different active components, and the different food sources. Particular interest was addressed to the metabolic syndrome, which is considered among the main causes of morbidity and mortality of populations both in industrialized areas and in developing countries. The authors have proposed both original studies and reviews of the literature data both reiterating the role of bioavailability (already mentioned above) and identifying the components that have been most active in the diseases considered (in addition to the metabolic syndrome in the various forms, Crohn’s disease, and anxiety disorders). The sources of polyphenols described include both plants already studied and appreciated for their beneficial activities (*Vaccinium macrocarpon* or cranberry; *Zea mais* or pigmented corn; *Tilia tormentosa* or linden) and plants or fruits less known in European culture but with promising future development (riceberry from Thailand, Maqui, etc.).
